# Unravelling Differential DNA Methylation Patterns in Genotype Dependent Manner under Salinity Stress Response in Chickpea

**DOI:** 10.3390/ijms24031863

**Published:** 2023-01-18

**Authors:** Khushboo Gupta, Rohini Garg

**Affiliations:** Shiv Nadar Institution of Eminence, NH-91 Tehsil Dadri, District Gautam Buddha Nagar, Greater Noida 201314, Uttar Pradesh, India

**Keywords:** DNA methylation, differential methylation, differential gene expression, salinity stress, bisulphite sequencing, chickpea

## Abstract

DNA methylation is one of the epigenetic mechanisms that govern gene regulation in response to abiotic stress in plants. Here, we analyzed the role of epigenetic variations by exploring global DNA methylation and integrating it with differential gene expression in response to salinity stress in tolerant and sensitive chickpea genotypes. Genome-wide DNA methylation profiles showed higher CG methylation in the gene body regions and higher CHH methylation in the TE body regions. The analysis of differentially methylated regions (DMRs) suggested more hyper-methylation in response to stress in the tolerant genotype compared to the sensitive genotype. We observed higher enrichment of CG DMRs in genes and CHH DMRs in transposable elements (TEs). A positive correlation of gene expression with CG gene body methylation was observed. The enrichment analysis of DMR-associated differentially expressed genes revealed they are involved in biological processes, such as lateral root development, transmembrane transporter activity, GTPase activity, and regulation of gene expression. Further, a high correlation of CG methylation with CHG and CHH methylation under salinity stress was revealed, suggesting crosstalk among the methylation contexts. Further, we observed small RNA-mediated CHH hypermethylation in TEs. Overall, the interplay between DNA methylation, small RNAs, and gene expression provides new insights into the regulatory mechanism underlying salinity stress response in chickpeas.

## 1. Introduction

Chickpea (*Cicer arietinum* L.) is a widely consumed legume plant that provides dietary proteins to animals and humans. However, chickpea production is hampered by various abiotic and biotic factors depending on the ecological region [1]. Among these factors, soil salinity decreases the production and productivity of chickpeas in arid and semi-arid ecological zones [2]. The response mechanism of a crop plant to salinity stress depends upon the salt concentration, genotype, soil type, and developmental stage. Different chickpea genotypes exhibit different response mechanisms to salinity stress; some have a tolerant phenotype, whereas some have a sensitive phenotype [2]. This variability in phenotypic traits may be attributed to epigenetic or genetic variations and different regulatory mechanisms involved [3,4].

DNA methylation is an epigenetic regulatory mechanism used by the plant to survive under different environmental cues [5]. It occurs via a chemical modification involving the addition of a methyl group to the cytosine base by methyltransferases (MTases). In plants, it occurs in CG, CHG, and CHH (where H can be any other base except guanine) sequence contexts. The DNA MTases can create a de novo methylation mark at unmethylated cytosine sites or maintain the preexisting cytosine methylation. Several studies in plants have revealed that different MTases are responsible for methylation in different sequence contexts [5]. The epigenome profiling in MTase mutants of Arabidopsis suggested that the maintenance of CG context methylation is mediated by MET1 (methyltransferase) and CHG context by CMT3 (chromomethylase). However, CHH context methylation is maintained by DRMs (domains rearranged methyltransferases 1 and 2) and CMT2 via RNA-dependent and RNA-independent pathways, respectively [6]. 

Several studies have shown genome-wide abiotic stress-induced DNA methylation profile changes in plants [7]. Studies have shown that transcriptional regulation of genes is associated with altered DNA methylation patterns during stress [8,9,10]. It has previously been demonstrated that changes in DNA methylation profiles during abiotic stress can cause phenotypic variability in plants [8]. The spread of DNA methylation from nearby transposons and other repeats can also cause promoter/gene body DNA methylation [11]. The low gene expression levels at the methylated regions in the gene body might be due to the inhibition of DNA Pol II activity and transcription [12]. The genomic regions having variation in methylation between different genotypes of the same species are referred to as epialleles [13]. The establishment and extent of the duration of stress memory responsible for the generation of epialleles is still a matter of investigation [14]. In *Arabidopsis thaliana*, under salinity stress, the changes induced in DNA methylation patterns were partially transmitted to the progeny, but if the progeny is not under stress, the epigenetic status is gradually lost [15]. However, the plants exposed to abiotic stresses were more adaptable to stress, and epigenetic memory may be transmitted to future generations [16]. 

To understand the epigenetic mechanism involving the interplay between DNA methylation, gene expression, and small RNAs in response to salinity stress, we investigated the DNA methylation patterns in two well-characterized chickpea genotypes with contrasting responses to salinity stress. We analyzed the patterns of DNA methylation in protein-coding genes (PCGs) and transposable elements (TEs) under control and salinity stress conditions. The differentially methylated regions (DMRs) under salinity stress was identified and correlated with differential gene expression to understand the possible role of DNA methylation in the regulation of expression of genes involved in salinity stress response. Small RNA (smRNA) sequencing data were integrated to identify the role of RNA-dependent DNA methylation in regulating salinity stress-induced methylation. Altogether, this study provides mechanistic insights into DNA methylation-mediated regulation of salinity stress-responsive genes and reveals candidate gene sets with genotype-specific DNA methylation patterns that might regulate salinity tolerance in chickpeas.

## 2. Results

### 2.1. Genome-Wide DNA Methylation Landscape in Chickpea Genotypes 

We investigated the global DNA methylation profiles in the roots of two genotypes of chickpea, ICC V2 (salinity sensitive, SS) and JG 62 (salinity tolerant, ST), under control and salinity stress. These genotypes have been well characterized for their contrasting response to salinity stress at different stages of development and used extensively for quantitative trait loci mapping and generation of biparental populations in previous studies [17,18]. In this study also, a slight decrease in root weight under salinity stress was observed in the SS genotype, whereas no significant change was detected in the ST genotype. Likewise, the median root length difference between control and salinity-stressed plants was less evident for the SS genotype as compared to the ST genotype (Figure S1A). 

**Figure 1:**
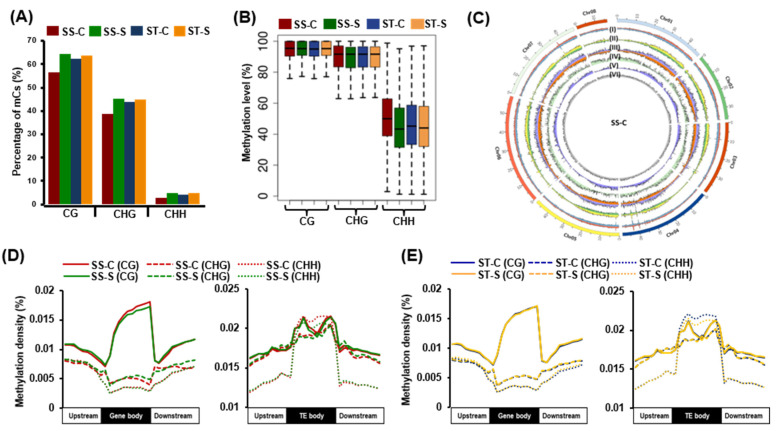
Whole genome DNA methylation profile in the chickpea genotypes. (**A**) Barplot showing percentage of methylcytosines (mCs) in different sequence contexts in control (SS-C, ST-C) and stress (SS-S, ST-S) samples of salinity-sensitive (SS) and salinity tolerant (ST) genotype. (**B**) Boxplot showing the distribution of methylation levels in each sequence context in different samples. (**C**) Circos plot depicting the chromosome-wide distribution of CG (I), CHG (II), CHH (III), genes (IV), TEs (V), and 24-nt smRNAs (VI) for reverse (bars above axis) and forward (bars below axis) strands, respectively, in a representative (SS-C) sample. (**D**,**E**) Methylation density in the gene body and 2 kb flanking regions, and TE body and 2 kb flanking regions in different sequence contexts in stress and control samples in the salinity-sensitive (SS-C and SS-S) (**D**) and salinity-tolerant (ST-C and ST-S) (**E**) genotypes.

We performed bisulphite sequencing of the genomic DNA isolated from the root samples of both the genotypes harvested under control and salinity stress conditions. In total, approximately 200–300 million high-quality reads representing ~30x sequencing depth were obtained that covered ~88% of the chickpea genome (Table S1). The highest fraction of methylated cytosines (mCs) was detected in CG (56.50–64.43%) context, followed by CHG (38.89–45.29%) and CHH (2.66–4.75%) contexts (Figure 1A). We observed a slightly higher fraction of mCs after stress treatment in both genotypes in all the sequence contexts. The average methylation level of mCs in the CG context (92.68–93.06%) was higher than CHG (87.53–88.23%) and CHH (44.94–51.11%) contexts (Figure 1B and Figure S1B). No significant differences in the methylation levels were observed between forward and reverse strands in all the samples (Figure S1C). Further, no significant change in the overall methylation levels in CG and CHG contexts between control (SS-C, ST-C) and stressed (SS-S, ST-S) samples of both SS and ST genotypes was observed. However, decreased methylation level was observed in the CHH context under salinity stress as compared to control in the SS genotype and marginally lesser in the ST genotype (Figure 1B). Global methylation patterns of mCs (per 100 kb on all the chickpea chromosomes) suggested higher CHH context methylation density in the chromosomal regions harboring a high density of TEs Figure 1C and Figure S1D). 

Next, we determined the distribution of DNA methylation in PCGs, TEs, and their 2 kb flanking regions in CG, CHG, and CHH contexts in control and stress samples of both genotypes. We observed that methylation density within the gene body regions was highest in the CG context, whereas it was higher in the flanking regions for CHG and CHH contexts. In addition, the methylation density in CG and CHH contexts at the transcription start sites (TSSs) and transcription termination sites (TTSs) was found to be lower as compared to the flanking regions. In contrast, the density of mCs in the TEs was highest in the CHH context compared to CG and CHG contexts (Figure 1D,E). 

### 2.2. Identification of Differentially Methylated Regions in Protein-Coding Genes and TEs 

**Figure 2:**
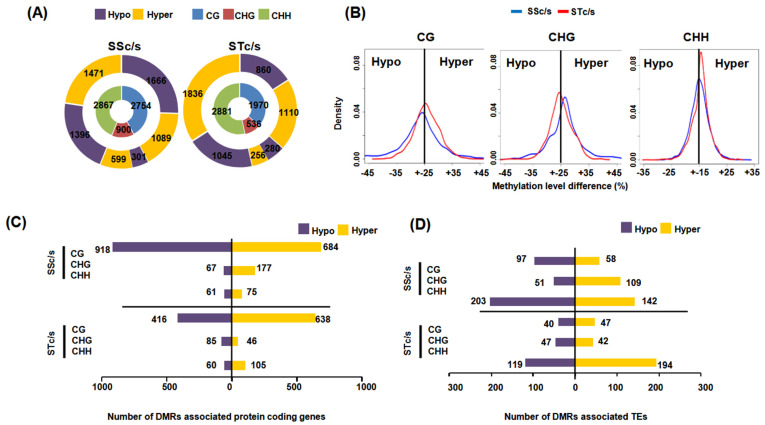
Identification and analysis of differentially methylated regions (DMRs). (**A**) Number of hypo and hypermethylated DMRs (outer circle) identified in different sequence contexts (CG, CHG, and CHH, inner circle) in the SS and ST genotypes under salinity stress. (**B**) Methylation level differences shown via kernel density plots in CG, CHG, and CHH sequence contexts. (**C**) Number of protein-coding genes associated with hypo and hypermethylated DMRs. (**D**) Number of TEs associated with hypo and hypermethylated DMRs. SSc/s and STc/s represent a comparison of salinity stress with control samples of SS and ST genotype, respectively.

To study the methylation dynamics under salinity stress in the SS and ST genotypes, differentially methylated regions (DMRs) between stress and control samples were identified in both genotypes. A total of 6521 DMRs were identified in the SS genotype (SSc/s), and a total of 5387 DMRs were identified in the ST genotype (STc/s) under salinity stress. The highest number of DMRs was identified in the CHH context (SSc/s; 2867, STc/s; 2881), followed by CG (SSc/s; 2754, STc/s; 1970) and CHG (SSc/s; 900, STc/s; 536) contexts under salinity stress (Figure 2A). Further, DMRs were categorized into hypomethylated and hypermethylated based on the methylation level differences in the stress samples as compared to the control sample. In the SS genotype, a greater number of hypomethylated DMRs (1666) were detected compared to the hypermethylated DMRs (1089) in the CG context. However, a higher number of CHG and CHH context hypermethylated DMRs (599 in CHG and 1471 in CHH) were obtained compared to hypomethylated DMRs (301 in CHG and 1396 in CHH). In the ST genotype, we observed more number of hypermethylated DMRs in CG (1110) and CHH (1836) contexts as compared to hypomethylated DMRs (860 CG and 1045 CHH), while an almost similar number of hyper (280) and hypomethylated (256) DMRs were obtained in the CHG context. In the CHH context, we observed more hypermethylation in both SS and ST genotypes under salinity stress (Figure 2A). Overall, we observed higher CG and CHH context DMR density in the ST genotype compared to the SS genotype (Figure 2B; Table S2). 

Further, to understand the influence of differential methylation on PCGs and TEs, we analyzed the presence of DMRs in the gene body/TE body and their flanking regions. We observed the highest number of CG context hyper and hypomethylated DMRs associated with PCGs in all the comparisons (Figure 2C). However, a greater number of CHH context DMRs were observed in the upstream regions of PCGs, and more CG context DMRs were associated with downstream regions of PCGs in all the comparisons (Figure S2A). Among the DMRs associated with TEs, a higher number of CHH DMRs were present within the TE body and their flanking regions (Figure S2B). In addition, a greater number of CHH context hypomethylated DMRs were associated with TEs in the SS genotype (Figure 2D and Figure S2B). Gene ontology (GO) enrichment analysis of the DMR-associated PCGs suggested their involvement in biological processes, such as ATPase activity, carboxylic acid metabolism, embryonic and seed development, transporter activity, response to abiotic stimulus, oxidoreductase activity, protein amino acid phosphorylation and response to hormones. A few genes involved in the flavonoid metabolic process, ion transport, and meristem structural organization were also associated with differential methylation between SS and ST genotypes. The genes involved in helicase activity, chromatin modification, and pigment biosynthesis were found to be differentially methylated in the ST genotype under salinity stress. However, genes involved in root hair differentiation, trichrome differentiation, and tRNA acylation were enriched among DMR-associated genes in the SS genotype (Figure S2C). 

### 2.3. Differential Gene Expression under Salinity Stress in the Chickpea Genotypes

Differential gene expression analysis was performed to study the transcriptional variations in the chickpea genotypes under salinity stress, followed by their correlation with DNA methylation status. The high-quality RNA-seq reads were mapped to the Kabuli chickpea genome (Table S3), and differentially expressed genes (DEGs) were determined. A total of 461 DEGs (159 upregulated and 302 downregulated) were identified in the SS genotype under salinity stress. Likewise, 743 DEGs (504 upregulated and 239 downregulated) were identified in the ST genotype under salinity stress (Figure S3A,B; Table S4). A total of 359 and 641 DEGs were found unique to SSc/s and STc/s, respectively. However, 102 DEGs were common between SSc/s and STc/s (Figure S3A). The DEGs showing up-regulation in both genotypes were involved in processes such as methyltransferase activity, malate transporter activity, and hormone biosynthetic process. However, commonly downregulated DEGs were enriched in pathways, such as aging, cation channel activity, and oxidoreductase activity (Table S5). In addition, we identified 1196 DEGs between the two genotypes under control conditions. Of these, 853 genes were differentially expressed under control conditions, while only 343 genes were differentially expressed in both (39) or in any one of the genotype (113 in SSc/s and 191 in STc/s) under salinity stress (Table S4). A similar pattern of differential gene expression has been reported in one of the previous study as well [17]. This analysis suggested that even though these genotypes have a large number of DEGs under control conditions, only a small fraction of these are affected by salinity stress in each genotype, and a larger gene set is differentially expressed in a genotype-dependent manner.

Further, to cluster the genes exhibiting similar expressions, K-means clustering analysis was performed on all the DEGs. We identified five clusters (clusters I to V) exhibiting preferential expression of genes in one or more sample/s. Cluster I harbored 229 genes downregulated in the SS genotype, cluster II contained 202 genes downregulated in the ST genotype, cluster III was comprised of 238 DEGs that were upregulated in the SS genotype, cluster IV (238 DEGs), and cluster V (195 DEGs) showed upregulation in the ST genotype (Figure S3C). GO enrichment analysis of these cluster-specific DEGs revealed transcripts involved in diverse processes. The genes involved in processes, such as regulation of transcription, hydrolase activity, oxidoreductase activity, kinase activity, and carboxylesterase activity, were represented in all the clusters (Figure S3D). The genes involved in pathways, such as photosynthesis and amino acid transport, were specific to cluster V, whereas genes associated with pathways, such as hydrogen symporter activity and flavonoid biosynthetic processes were specific to cluster III. The genes involved in UDP glycosyltransferase activity towards flavonoids were specific to clusters I and II. The genes involved in cell death were found to be enriched in clusters III and IV. The genes involved in metal ion transport were downregulated in both SS and ST genotypes. Overall, gene expression analysis suggested differential transcriptional regulation of several stress-responsive genes under salinity stress in the SS and ST genotypes.

### 2.4. Influence of DNA Methylation on Differential Gene Expression and Co-Relation between Different Methylation Contexts 

To study the influence of DNA methylation on gene expression, we categorized PCGs into five classes based on their expression level. We observed a positive correlation between CG context DNA methylation in the gene body and gene expression, wherein the genes with higher expression showed higher CG methylation density in the gene body regions. However, a negative correlation between methylation density at the TSS and TTS sites in all the sequence contexts and gene expression was observed, suggesting that methylation at these sites contributes to lower gene expression (Figure S4A). Overall, a similar relationship between DNA methylation and gene expression was observed in both SS and ST genotypes under control conditions (Figure S4A). Further, the genes with very low expression levels exhibited higher CHG and CHH methylation as compared to highly expressed genes, suggesting that lowly expressed genes have high methylation density as well as methylation levels (Figure S4B). 

**Figure 3:**
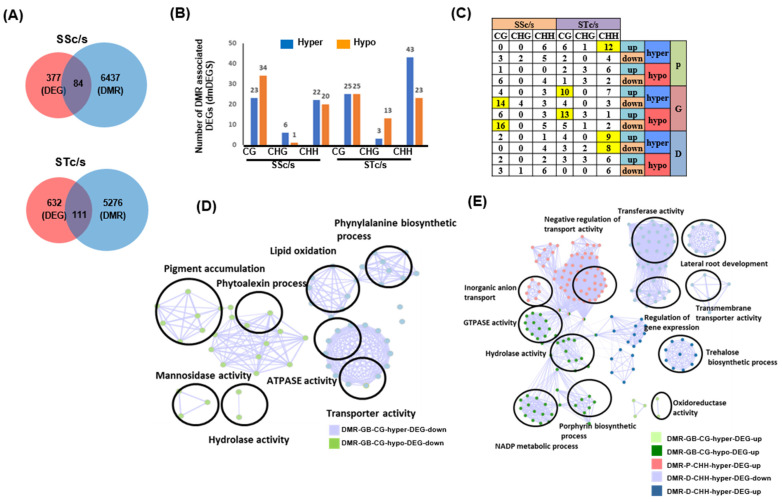
Association of differentially methylated regions with differentially expressed genes. (**A**) Venn plots depicting the number of differentially methylated regions and differentially expressed genes in salinity sensitive (SSc/s) and salinity tolerant (STc/s) genotypes under salinity stress. (**B**) Number of DEGs associated with hyper and hypomethylated DMRs present in the gene body and flanking regions in different sequence contexts. (**C**) Distribution of up and down-regulated dmDEGs harboring hypo or hypermethylated DMRs in different genic regions [promoter (P), gene body (GB) and downstream (D)] in SSc/s and STc/s. Yellow highlighted cells represent enriched sets. (**D**,**E**) Enrichment analysis of highlighted dmDEGs in SSc/s (**D**) and STc/s (**E**) genotypes under salinity stress.

Next, to understand the role of DNA methylation in the regulation of gene expression under salinity stress, we analyzed the relationship between DEGs and DMRs. We found 84 DEGs associated with DMRs (dmDEGs) in the SS genotype. Likewise, 111 dmDEGs were identified in the ST genotype under salinity stress (Figure 3A). The distribution of hypo and hypermethylated dmDEGs in different contexts (CG, CHG, and CHH) suggested a greater number of DEGs associated with CG context hypomethylated DMRs in the SS genotype and a greater number of hypermethylated CG DMRs were found associated with DEGs in the ST genotype (Figure 3B). Further, a higher number of DEGs was associated with hypermethylated CHH DMRs in the ST genotype (Figure 3B).

These DMRs were further categorized for their presence in the flanking and gene body regions of DEGs (Figure 3C, Table S6). This analysis revealed a higher number of downregulated DEGs (30) harboring CG methylation in their genic regions in the SS genotype. However, in the ST genotype, more enrichment of upregulated DEGs (23) harboring CG methylated DMRs within their gene body was observed. In addition, we observed a significant number of upregulated genes harboring hyper CHH methylation in their upstream (12) and downstream (9) regions in the ST genotype (Figure 3C). Further, variation in methylation levels was observed in the CG context, majorly in the gene body and the CHH context in the flanking regions of DEGs in both SS and ST genotypes (Figure S4C,D). 

Next, we analyzed the relationship between the direction of differential methylation and differential gene expression of dmDEGs. A negative correlation between CHG methylation and gene expression was observed in the ST genotype (STc/s) under salinity stress. However, no conclusive relationship between CG and CHH context DMRs with the level of gene expression was observed (Figure S5A). The GO enrichment analysis of CG dmDEGs suggested that genes involved in processes, such as GTPase activity, hydrolase activity, response to stress, and carbohydrate catabolic processes, were enriched in the ST genotype. However, genes involved in transmembrane transporter activity, oxidoreductase activity, phytoalexin metabolic processes, and pigment accumulation were enriched in the SS genotype (Figure S5B). Similarly, CHH dmDEGs showed enrichment of biological processes, such as transporter activity, auxin homeostasis, lipid transport, methyltransferase activity, and secondary metabolite biosynthesis in the ST genotype. The CHH dmDEGs in the SS genotype were enriched in biological processes, such as cation binding, dehydrogenase activity, and amino acid binding (Figure S5C). The GO enrichment analysis of significantly enriched CG dmDEGs in the SS genotype revealed their involvement in transporter activity, lipid oxidation and phenylalanine biosynthesis, pigment accumulation, phytoalexin, and mannosidase activity (Figure 3D). In the ST genotype, dmDEGs showing downregulated gene expression and harboring CHH context hypermethylation in the downstream regions were involved in the processes, such as lateral root development and trehalose biosynthetic process. The upregulated dmDEGs harboring gene body hypomethylation in the CG context were involved in GTPase activity, hydrolase activity, and NADP metabolic process; however, those having hypermethylation were involved in oxidoreductase activity (Figure 3E). In addition, dmDEGs associated with CHH hypermethylation in their promoter regions were found to be involved in the regulation of transport activity. 

The integrative genome views (IGV) of DMR regions and differential gene expression of several dmDEGs (Figure S5D) in the SS genotype, including *Ca_12185* and *Ca_02410,* showed gene body CG hypermethylation and downregulated gene expression. Similarly, dmDEGs, *Ca_12433* and *Ca_09442*, harbored gene body CG hypomethylation in the SS genotype. The *Ca_02137* displayed CHH hypermethylation in the downstream region. The genes, *Ca_20130* and *Ca_25894*, showed hypomethylation in the CG context in their gene body region, and *Ca_12085* had hypermethylation in the CHH context in its promoter region, and these genes were upregulated in the ST genotype under salinity stress. The gene *Ca_13125* harbored gene body CG hypermethylation and was upregulated in the ST genotype. Overall, our analysis identified candidate dmDEGs methylated in a genotype-dependent manner under salinity stress and might be involved in the regulation of root traits in the chickpea genotypes. 

To understand the role of known DNA MTases in salinity stress response, their expression levels were analyzed in all the samples. The *CaCMT1*, *CaCMT2*, *CaDMR1,* and *CaDRM2* were expressed at higher levels in the ST genotype as compared to the SS genotype under salinity stress (Figure S6A). The CMT2, DRM 1, and DRM2 are known to regulate CHH context methylation guided by the action of small RNAs [19]. Higher DRM expression levels under salinity stress might be responsible for higher CHH hypermethylation in the ST genotype compared to the SS genotype (Figure 2B). DNA methylation levels are regulated by other proteins, such as demethylases, Argonaut, and RNA-dependent RNA polymerase genes. The two DNA demethylase gene homologs, *DME* and *ROS1*, showed lower expression in both SS and ST genotypes under salinity stress, while *DCL*, *AGO,* and *RDRP* homologs showed no significant change in expression in the SS genotype but downregulation of *DCL3*, *DCL4*, *DCL1*, *AGO2* and *AGO6* in the ST genotype was observed under salinity stress. Interestingly, AGO4 homologs known to regulate CHH and CHG methylation in Arabidopsis [20] showed higher expression in the ST genotype under salinity stress, suggesting the role of smRNA-mediated regulation of gene expression under salinity stress in the ST genotype (Figure S6B). The methylation in the CG context is mediated predominately by MET1 [5]; however, we did not observe any difference in *MET1* expression under salinity stress in both genotypes. 

Recently, the role of CMT3-mediated gain in gene body CG methylation was observed in *Eutrema salsugineum* [21]. To understand the correlation between CG, CHG, and CHH contexts, the methylation level of dmDEGs was assessed in the gene body and flanking regions. A high correlation of CG context hyper and hypomethylation within the gene body with CHG and CHH context methylation was observed in both ST and SS genotypes under salinity stress (Figure S6C). In contrast, less correlation of gene body CG DMRs was observed with their flanking region CG, CHG, and CHH methylation levels (except for gene body hyper CG DMR in the SS genotype under control conditions). Similarly, flanking region CG DMRs showed a correlation with flanking region CHG and CHH DMRs, but not with gene body DMRs (Figure S6D). Altogether, a positive correlation between gene body CG, CHG, and CHH methylation and flanking region CG, CHG, and CHH methylation was observed in the DMRs in SSc/s and STc/s (Figure S6D). Overall, this observation suggests that CHG and CHH methylation might play a role in mediating CG methylation [22].

### 2.5. Small RNA-Mediated DNA Methylation Dynamics in TEs 

**Figure 4:**
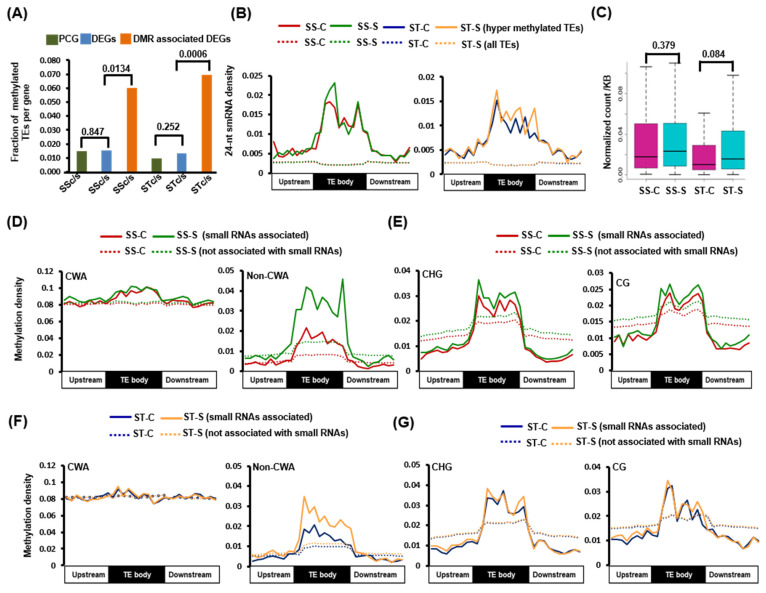
Influence of small RNAs on TE methylation. (**A**) Frequency of TEs within protein-coding genes, differentially expressed genes (DEGs), and dmDEGs, numbers on top represent significance value calculated by fisher exact test. (**B**) 24-nt small RNA density in CHH-hypermethylated TEs and all TEs in control (SS-C, ST-C) and stress (SS-S, ST-S) samples of both genotypes. (**C**) Normalized count of smRNAs associated with hypermethylated TEs in CHH context per kb of TE in SS-C, ST-C, SS-S, and ST-S samples (significance is calculated by Wilcoxon test). (**D**–**G**) Methylation density of CHH hypermethylated TEs in CWA-CHH sub-context and non-CWA-CHH sub-context that are associated and not associated with 24-nt smRNAs in SSc/s (**D**) and STc/s (**F**). (**E**,**G**) CHG and CG context methylation density of CHH hypermethylated TEs that are associated and not associated with 24-nt smRNAs in (**E**) SSc/s and (**G**) STc/s.

To understand the role of TE methylation in the regulation of gene expression, we estimated the frequency of methylated TEs in all the PCGs, DEGs, and dmDEGs. We observed a higher frequency of methylated TEs in dmDEGs compared to all PCGs and all DEGs in both SS (6 times higher) and ST (7 times higher) genotypes under salinity stress. However, no significant difference in TE frequency between PCGs and DEGs in both genotypes was observed (Figure 4A). This observation suggested that the association of methylated TE with dmDEGs might contribute to differential methylation and differential expression of these genes under salinity stress. Similarly, the frequency of all TEs was also higher in DEGs and dmDEGs in both genotypes. The dmDEGs having a higher frequency of TEs were involved in pathways, such as UDP-glucosyltransferase activity, regulation of transcription, DNA binding, and response to stimulus in the SS genotype. However, dmDEGs of the ST genotype were associated with ATPase activity, photosynthesis, hydrolase activity, regulation of gene expression, and response to abiotic stimulus processes. We observed the highest number of CHH DMRs within the TE body and flanking regions (Figure 2D and Figure S2B), which could potentially be mediated by smRNAs via the RNA-directed DNA methylation pathway in plants [23,24]. 

To understand the influence of smRNAs on the methylation of TEs, we investigated the distribution of smRNAs and methylation density in the TEs and their 2 kb flanking regions. The small RNA sequencing followed by size distribution analysis of the identified smRNAs suggested the highest fraction of 24-nt smRNAs in all the four samples analyzed (Table S7; Figure S7A). We observed that the smRNA density (24-nt and 21-nt) was higher in the CHH hypermethylated TEs as compared to all TEs in both SS and ST genotypes (Figure 4B and Figure S7C). However, no significant change in smRNA density was observed in CG and CHG hypermethylated TEs compared to all TEs (Figure S7D). The 24-nt smRNAs in CHH hypermethylated TEs exhibited higher expression in the stress samples of both genotypes (Figure 4C). Further, CHH context mCs were categorized into different sub-contexts, such as CWA (CTA and CAA) and non-CWA contexts, as it is known that CMT2 mediate methylation in CWA context and DRMs methylate in non-CWA context, respectively, to establish de novo methylation [25,26]. Higher non-CWA context methylation density was observed in the hypermethylated TEs associated with smRNAs compared to hypermethylated TEs not associated with smRNAs. Further, the density of non-CWA methylation in hypermethylated TEs associated with smRNAs was higher in the stress samples in both SS and ST genotypes (Figure 4D,F). However, no significant difference in the methylation density of CG and CHG context mCs in the hypermethylated TEs associated with smRNAs between control and stress samples was observed (Figure 4E,G). In addition, no significant difference in smRNA and methylation densities was observed between the CHG and CG hypermethylated TEs harboring smRNAs and those not harboring smRNAs (Figure S7C,D). Similarly, higher 21-nt smRNA density was detected for CHH hypermethylated TEs, and higher non-CWA methylation density in the TEs associated with 21-nt smRNAs under salinity stress was observed (Figure S7E,F). These results suggest a possible role of 24-nt and 21-nt smRNAs in stress-induced hypermethylation in the CHH context in TEs on non-CWA (CHH) and regulation of gene expression. The base frequency distribution of 24-nt smRNAs associated hypermethylated TEs showed enrichment of CT motif at the 3′-end (Figure S7B), which correlates with AGO4 protein signature on 24-nt smRNAs reported in the previous study [27], suggesting the role of AGO4-24nt smRNAs in hypermethylation of TEs.

### 2.6. Identification of Conserved and Specific DMR-Associated DEGs (dmDEGs) between Genotypes 

**Figure 5:**
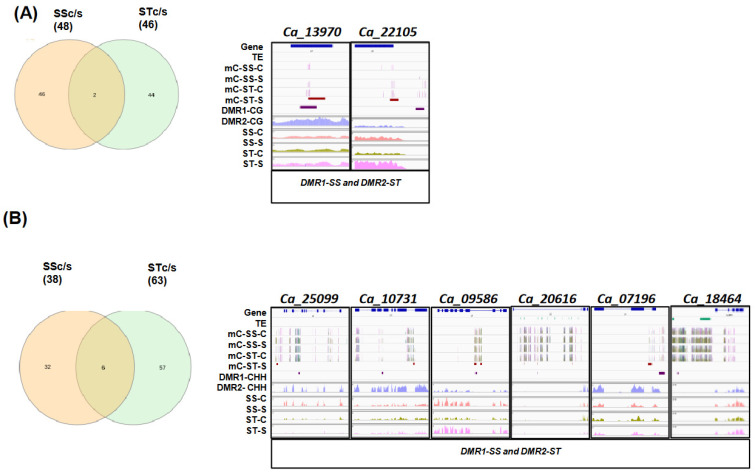
Genes displaying differential methylation and differential gene expression in both genotypes under salinity stress. (**A**,**B**) Venn plot and IGV plots representing common CG-dmDEGs (**A**) and CHH-dmDEGs between SSc/s and STc/s (**B**) in response to salinity stress. SSc/s and STc/s represent a comparison of salinity stress with control samples of SS and ST genotypes. Each view includes the gene and TE location (rows 1 and 2) on the selected view, followed by the position of methylated cytosines (mCs in CG-purple, CHG-teal, and CHH-olive green, context) in the top panel (rows 3, 4, 5, and 6), differentially methylated regions (DMR1-SSc/s, DMR2-STc/s) in the gene body or flanking region (rows 7 and 8) and gene expression in control (SS-C, ST-C) and stressed (SS-S and ST-S) samples of both genotypes in the last four rows (row 9, 10,11 and 12).

Further, to identify the dmDEGs that were common between different comparisons in CG, CHG, and CHH contexts, we compared dmDEGs of SSc/s and STc/s. Two dmDEGs identified in both SS and ST genotypes under stress, *Ca_13970,* and *Ca_22105*, showed differential CG methylation in their gene body and promoter regions (Figure 5A). *Ca_13970* encodes a homolog of the Arabidopsis transmembrane protein, which is known to be involved in oxidative stress and osmotic stress responses [28]. *Ca_22105*, a homolog of AT2G19330, encoding PIRL6, a member of the plant intracellular Ras-group-related LRRs (leucine-rich repeat proteins) and was shown to be alternatively spliced and regulate gametophyte development [29].

In the CHH context, six dmDEGs were common between SS and ST genotypes (Figure 5B). The gene *Ca_25099* encoding a member of the MATE efflux family protein FRD3 was downregulated under salinity stress in both genotypes with a gain of gene body and promoter methylation. In a previous study, *FRD3* mutants showed leaf chlorosis, plant growth reduction, Fe accumulation in the root vascular tissues, and alterations in Fe, Mn, and Zn homeostasis [30]. Similarly, the *Lox* gene homolog, *Ca_10731*, was downregulated and hypermethylated in both SS and ST genotypes under stress, suggesting stress-induced methylation and regulation of gene expression. The gene *Ca_09586*, upregulated and hypermethylated in both genotypes under stress, encodes a homolog of myo-inositol-1-phosphate synthase isoform (MIPS). The overexpression of this gene is known to enhance salinity tolerance in poplar plants [31]. The gene *Ca_20616*, a bHLH homolog, was downregulated in both genotypes and hypermethylated in the SS genotype and hypomethylated in the ST genotype, while another bHLH homolog, *Ca_07196*, was downregulated and hypermethylated in both the genotypes. The homologs of these genes are known to be involved in the regulation of iron transport in roots [32]. However, gene *Ca_18464* was hypomethylated in both genotypes and upregulated in the SS genotype while being downregulated in the ST genotype. This gene has no Arabidopsis homolog and may represent a legume-specific gene regulated by salinity stress. 

Interestingly, a major fraction of dmDEGs was found specific to the SS (46-CG and 32-CHH) or ST (44-CG and 57 CHH) genotypes. The subset of dmDEGs that harbors CG-DMRs in the SS genotype was involved in processes such as senescence, amino acid transport, calcium transmembrane transport, lipid oxidation, mannose metabolic process, anthocyanin accumulation, pectate lyase activity, mandelonitrile lyase, while those of ST genotype are involved in processes such as senescence, vascular tissue pattern formation, regulation of stomatal movement, glutathione catabolic process, fatty acid beta-oxidation, pentose phosphate shunt, sterol biosynthetic process, maltose biosynthetic process, chromatin binding, copper ion binding. Similarly, CHH-dmDEGs of the SS genotype were involved in ABA signaling, senescence, proline dehydrogenase activity, steroid dehydrogenase activity, and glutamate biosynthetic processes, while those of the ST genotype were enriched in terms such as lignin biosynthetic process, trehalose biosynthetic process, inositol triphosphate metabolic process, lateral root formation, and negative regulation of anion channel (Table S8). Some of these genes seem to be methylated in a genotype-dependent manner under salinity stress (Figure S5D,E). Overall, these DMR-DEGs, which are specific to the ST genotype, represent important candidate target genes that might be regulated by differential methylation and contribute to salinity stress tolerance.

## 3. Discussion

In this study, we sought to understand the role of DNA methylation in salinity stress response in chickpeas. Recent studies have shown evidence in favor of epigenetic regulation influencing the stress response in plants through the regulation of gene expression [33]. Due to the advancement in sequencing technologies, various genomic resources of chickpeas, such as reference genome, transcriptome, non-coding RNAs, and genetic variations, are available [4,34,35,36,37]. However, the dynamics and relationship between DNA methylation and gene expression for stress adaptation have been rarely studied in this crop plant. Therefore, it is important to understand the epigenetic regulation of salinity stress response in different genotypes in chickpeas. 

In the current study, we employed single-base resolution DNA methylation profiling along with gene expression analysis to decode the role of DNA methylation in salinity stress response in chickpeas. We observed the highest fraction of mCs in the CG context, followed by CHG and CHH contexts in both SS and ST genotypes. The average methylation level was highest in the CG context, followed by CHG and CHH, which have been reported in previous studies, too [38,39]. The density of mCs in the gene body region was found to be highest in the CG context, whereas the density was highest in the CHH context for the TE body, as has been reported in many plant species [10,38]. A slight peak at the transcription start site (TSS) and transcription end site (TTS) of mCs in the CHG context was observed, which could modulate nucleosome occupancy and gene transcription [40]. The identification of DMRs enabled the identification of genotype-specific methylated regions. More CG context hypermethylation was observed in the ST genotype, whereas more hypomethylation was observed in the SS genotype under salinity stress. However, hypermethylation in the CHH context was observed in both genotypes. These results suggested the overall hypermethylation in CG and CHH contexts and CHG hypomethylation in the ST genotype in response to salinity stress. The association of differential DNA methylation regions revealed an association of CG context DMRs with PCGs and an association of CHH context DMRs with TEs. Many studies have demonstrated the role of differential methylation associated with stress response mechanisms in plants [41,42].

The gene expression profiling in SS and ST genotypes in response to salinity stress revealed more upregulated DEGs in the ST genotype and more downregulated DEGs in the SS genotype. The cluster of upregulated DEGs in the ST genotype under stress showed enrichment of laccase activity. The overexpression of the rice *OsChI1* gene (a putative laccase precursor) in Arabidopsis enhanced tolerance to drought and salinity stresses [43]. The pathways, such as the flavonoid biosynthetic process specifically detected in cluster III, have also been reported to be heavily influenced by salinity stress [44]. The upregulated DEGs in both genotypes (cluster V) involved in photosynthesis have been reported to protect the photosystem by enhancing the photosynthetic performance in sorghum [45]. Further analysis revealed a positive correlation between gene body CG context DNA methylation and gene expression. However, a negative correlation at TSS and TTS sites suggested that DNA methylation at gene ends is associated with the repression of gene expression [10,12]. The relationship between differential methylation and differential gene expression under salinity stress showed DEGs associated with hypermethylated DMRs in the ST genotype. A similar pattern of hypermethylation under salinity has already been reported in other studies, too [46,47]. These results suggest hypermethylation in chickpeas might be associated with the ST genotype in response to salinity stress. Further, GO enrichment analysis in the SS genotype resulted in the identification of dmDEG, *Ca_02410*, a homolog of *AT4G23030* (MATE efflux family protein), expressed in roots, which is known to be involved in iron homeostasis, aluminum tolerance, efflux of xenobiotics, and regulation of local auxin biosynthesis [48]. Other downregulated dmDEGs, *Ca_12433* and *Ca_09442*, represented homologs of Arabidopsis genes *AT2G40410* (*AtCAN2*) and *AT2G38470* (*AtWRKY33*). A mutation in *AtCAN2* resulted in better tolerance to salt stress and a lower level of H_2_O_2_ accumulation [49], while mutations in *WRKY33* inhibited root growth under salt stress [50]. *Ca_02137*, a homolog of *AT4G37070* (phospholipase-A IVA), is downregulated in the ST genotype under salinity stress and displayed CHH hypermethylation in the downstream region. The downregulation of Arabidopsis patatin-related phospholipase A was shown to reduce lateral root development [51]. Another upregulated gene, *Ca_12085*, with promoter CHH hypermethylation, encodes a Pht1:5 phosphate transporter and is known to inhibit primary root growth when overexpressed [52]. These dmDEGs showing differential DNA methylation in different sequence contexts and gene expression under salinity stress in chickpeas can serve as potential candidate genes for validating their role in salinity stress tolerance. 

The gene expression analysis of DNA MTases suggested lower expression of *CMT* and higher expression of *DRM1* and two in the ST genotype and *DRM2* in the SS genotype under salinity stress. However, the expression level of MET1 decreased under stress, suggesting the role CMTs or DRMs in mediating CG gene body methylation, as reported in a few other studies [19,21]. A higher correlation between the methylation levels of hyper and hypomethylated CG DMRs was observed with gene body CHG and CHH context methylation in both the genotypes, suggesting the possible role of non-CG context gene body methylation in mediating gene body CG context methylation under salinity stress. 

A higher frequency of methylated TEs in dmDEGs in both the genotypes as compared to DEGs and PCGs suggested a possible role of TEs in differential methylation and differential gene expression. Further, a higher number of CHH DMRs were associated with TEs, which could be initiated by small RNAs. A higher density of smRNAs was observed in these methylated TEs compared to all TEs. Higher mCs density in non-CWA (CHH) sub-context in TEs suggested the probable role of smRNAs in mediating methylation in the CHH context under salinity stress in both genotypes. 

## 4. Conclusions

In conclusion, single-base resolution DNA methylation maps of two chickpea genotypes under control and salinity stress conditions have been generated. Overall, hypermethylation was observed in the ST genotype under salinity stress. The in-depth analysis of methylation patterns revealed a correlation between several differentially expressed genes with differential methylation. Few of the differentially expressed genes were found regulated specifically in only one genotype, while few genes were commonly regulated under stress conditions in both genotypes. More DEGs were associated with CG and CHH DMRs than CHG DMRs. A higher fraction of dmDEGs showed an association with hyper CHH context DMRs in the ST genotype compared to the SS genotype, which could be mediated by higher DRM expression in the ST genotype. These CHH-dmDEGs were found to be associated with pathways, such as lateral root development, trehalose biosynthetic process, regulation of membrane transport, and gene expression regulation in the ST genotype. Further, CG hyper-methylation in the gene body region correlated with the gene body CHG and CHH methylation. Our analysis suggested that small RNA-mediated TE methylation in the CHH context under salinity stress could play a role in differential gene expression. The regulation of gene expression via differential methylation patterns revealed several genes that can serve as potential targets for epigenetic modification-based gene regulation and salinity stress tolerance in plants. 

## 5. Material and Methods

### 5.1. Plant Materials and Genomic DNA Isolation 

Two chickpea genotypes (*Cicer arietinum* L.), ICC V2 (salinity sensitive, SS), and JG 62 (salinity tolerant, ST), were used in this study. For salinity stress, 12-day-old plants were given 80 mM of NaCl for 6 days on alternating days, while control plants were watered with RO water. The roots of stress-treated and control plants were harvested on the 18th day of growth for root length and root weight measurements or snap-frozen in liquid nitrogen and stored at −80 ℃ until further use. This experimental setup was repeated to collect multiple independent biological replicates. Genomic DNA was isolated using Qiagen DNeasy Minikit (Qiagen) as per the manufacturer’s instructions. 

### 5.2. Whole-Genome Bisulphite Sequencing and Data Analysis 

Bisulphite sequencing library preparations were conducted using the manufacturer’s protocol as described in a previous study [53]. The qualified libraries were sequenced to generate 90 bp paired-end reads to obtain ~30× coverage for each sample. The raw reads were processed for adaptor sequence and low-quality reads removal using the NGSQC toolkit (v2.3.3). The filtered reads were aligned to the Kabuli chickpea genome (V1.0) [35] using Bismark (v0.14.3) [54], and only the reads mapped at the unique positions were retained. The read alignment was also performed on the chickpea chloroplast genome to calculate the bisulphite conversion efficiency and error rate. The mCs in each sample were identified using methylKit (v1.5.3) based on a *p*-value cut-off of ≤0.0001 and minimum read coverage of 3. Differential methylation between the salinity stress and control conditions for each genotype was also identified using methylKit [55]. The 100 bp bins with at least 3 cytosine sites that are covered by ≥5 reads and a methylation level difference of 25 % (CG and CHG context) or 15 % (CHH context) with q-value cut off ≤ of 0.01 were considered to be DMRs as described in a previous study [10]. The methylation sub-context was identified using the “bismark_methylation_extractor” on the bismark output file. The distribution of mCs within the gene body/TE body and 2 kb flanking regions was conducted using in-house scripts based on the mid-position of the DMRs. Visualization of DNA methylation on the chickpea genome, along with genes, TEs, and smRNAs, was performed via Circos plots. The distribution of mCs, expression profiles, and DMR regions was visualized using Integrative Genome Viewer (v2.4.14). Correlation analysis between the methylation levels of CG, CHG, and CHH context was performed via the Pearson correlation coefficient method using ggplot2 in R. 

### 5.3. RNA Sequencing and Data Analysis 

The total RNA for all the samples (same as that used for bisulphite sequencing) was extracted using TRI Reagent (Sigma-Aldrich, St Loius, MO, USA), according to the manufacturer’s instructions. For sequencing, cDNA libraries were prepared for each sample, and sequencing was performed on the Illumina platform to generate 49 bp single-end reads. Quality control analysis was performed on the fastq files using NGS QC Toolkit. TopHat (V2.1.1) software was used to map the filtered reads on the Kabuli chickpea genome (V1.0). The differential expression of genes between control and salinity stress samples was determined by Cuffdiff (V2.1.1). The transcripts with *p*-value ≤ 0.05 and fold change of ≥1.5 and ≤−1.5 were considered to be significantly differentially expressed genes. Gene ontology enrichment analysis of DEGs and dmDEGs was performed using the BiNGO and enrichment tool from Cytoscape.

### 5.4. Small RNA Sequencing and Data Analysis 

Total RNA from all the samples was used to prepare small RNA sequencing libraries per the manufacturer’s recommendations (Illumina technologies). Sequencing for each sample was performed in single-end sequencing mode to obtain 49 bp long reads. Raw data files were processed to remove adapters using Cutadapt. High-quality reads representing the same sequences were collapsed into unique read(s). The frequency of these unique reads was used to determine the abundance of small RNAs. Bowtie (v1.1.2) mapping tool was used to map the reads to the chickpea reference genome (V1), allowing no mismatch. The reads mapped to non-coding sequences, structural RNAs (rRNA, tRNAs, and snoRNAs), and the chickpea chloroplast genome was removed as described in a previous study [10]. The 21-nt and 24-nt long reads representing small RNAs were selected for further analysis.

## Data Availability

Bisulphite sequencing data, RNA sequencing data, and small RNA sequencing data have been submitted to NCBI’s Gene Expression Omnibus (GEO) with the accession number GSE204730.

## Supplementary Materials

The following supporting information can be downloaded at: https://www.mdpi.com/article/10.3390/ijms24031863/s1.
