# Supplementary material for: Unravelling Differential DNA Methylation Patterns in Genotype Dependent Manner under Salinity Stress Response in Chickpea
Source: Int J Mol Sci. 2023 Jan 18;24(3):1863. doi: 10.3390/ijms24031863 (PMC9915442; doi:10.3390/ijms24031863)
Supplement: Supplementary file 1 [file ijms-24-01863-s001.zip › salinity-supplimentary-tables.pdf]

Table S1: Details of bisulphite sequencing libraries generated in this study.

		SS-C	SS-S	ST-C	ST-S
Total reads	Rep I	216517166	215614008	215443642	214927470
	Rep II	200244584	290959084	301267100	301693478
High quality reads	Rep I	216517130	215613986	215443622	214927442
	Rep II	200242704	290958822	301266918	301693294
Uniquely mapped read pairs	Rep I	55945579	57634013	56144344	55728169
	Rep II	44403836	74174529	68183504	70791619
Mapping efficiency (%)	Rep I	51.70%	53.50%	52.10%	51.90%
	Rep II	44.40%	51.00%	45.30%	46.90%
Genome coverage (%)	Rep I	88.43%	88.35%	88.54%	88.34%
	Rep II	88.29%	88.91%	88.61%	88.86%
Chloroplast DNA conversion rate (%)	Rep I	99.992	99.993	99.993	99.993
	Rep II	99.990	99.992	99.993	99.993
Error rate	Rep I	0.00782	0.00698	0.00672	0.00711
	Rep II	0.00978	0.00821	0.00744	0.0075
Total methylated Cs	Rep I	130236897	129970400	130471865	128587239
	Rep II	129614244	133485545	131990475	133475645
Total methylated C's (%)	Rep I	16.81	17.33	17.60	17.62
	Rep II	13.44	18.12	16.73	18.57

Table S3: Total number of raw reads generated, high quality reads and mapping percentage of RNA sequencing data in control and stress samples of SS and ST genotype.

	QC			Mapping	
	Total No. of reads	Total No. of HQ reads	% of HQ reads	Mapped reads	Over all mapping rate
SS-C1	11822910	11779659	99.63%	11555946	98.1%
SS-C2	12656423	12614898	99.67%	12373472	98.1%
SS-S1	10361560	10325023	99.65%	10123631	98.1%
SS-S2	10953338	10917290	99.67%	10717801	98.2%
ST-C1	8279106	8252039	99.67%	8093207	98.1%
ST-C2	11752512	11713945	99.67%	11407610	97.4%
ST-S1	10625852	10586951	99.63%	10385638	98.1%
ST-S2	11114251	11079906	99.69%	10870826	98.1%

Table S7: Total number of 21-nt and 24-nt small RNAs identified in control and stress samples of SS and ST genotype in response to salinity stress.

	21-nt	24-nt
SS-C	90160	1551008
SS-S	68028	1436690
ST-C	81835	1294844
ST-S	52378	724464
