# Supplementary material for: Unravelling Differential DNA Methylation Patterns in Genotype Dependent Manner under Salinity Stress Response in Chickpea
Source: Int J Mol Sci. 2023 Jan 18;24(3):1863. doi: 10.3390/ijms24031863 (PMC9915442; doi:10.3390/ijms24031863)
Supplement: Supplementary file 1 [file ijms-24-01863-s001.zip › Supplementary Information-details.pdf]

Supplementary Figure Legends

Fig. S1: Methylation levels in different sequence context. (A) Violin plots depicting effect of salt stress on root length and root weight in salinity sensitive (SS) and salinity tolerant (ST) genotype under control (SS-C and ST-C) and salinity stressed (SS-S and ST-S) conditions. (B) Fraction of methylated cytosines showing varying methylation level in CG, CHG and CHH sequence context in all four samples. (C) Box plots showing methylation levels in forward and reverse strands in three different sequence context in SS-C and ST-C, SS-S and ST-S samples. (D) Circos plots for SS-S, ST-C and SS-S samples, depicting chromosome wise distribution of CG (I), CHG (II), CHH (III), gene (IV), TEs (V), and 24-nt smRNAs (VI) for reverse (above axis) and forward (bars below axis) strand respectively.

Fig. S2: DMRs associated with flanking regions of protein coding gene and TEs. (A-B) Number of hypo and hyper methylated DMRs-associated with (A) protein coding genes and (B) TEs in promoter and downstream regions in SSc/s and STc/s comparisons under salinity stress response. (C) Gene ontology enrichment of DMRs associated with protein coding genes in SSc/s and STc/s comparisons.

Fig S3: Differential gene expression under salinity stress in two chickpea genotypes. (A) Venn diagram showing differentially expressed genes (DEGs) under salinity stress in SSc/s and STc/s comparisons. (B) Bar graph showing number of up and down regulated DEGs in each comparison. (C) Heatmap showing normalized expression of DEGs categorized into different cluster (I to V, based on K-means clustering). Scale at the bottom represents Z-score (pink represents low expression, green represents higher expression). (D) Gene ontology enrichment of DEGs of each cluster.

Fig S4: DNA methylation dynamics of genes expressed at different levels (A) DNA methylation of all protein coding genes expressed at different levels {expression = 0 FPKM (no expression), < 1FPKM (very low), >=1 to 5 FPKM (low), >=5 to 25 FPKM (moderate) and >25 FPKM (high)} in control and stress samples (SS-C, ST-C, SS-S and ST-S); (B) Heatmap representing gene expression and methylation level of all protein coding genes in SSc/s and STc/s comparisons. (C, D) Heatmap representing gene expression and methylation level of dmDEGs in (C) SSc/s and (D) STc/s comparisons. Scale at the bottom of the figure shows differential gene expression in form of log2 fold change; teal color shows down regulation and red color represents up regulation and for % methylation level (% ML); yellow represents lower methylation levels and pink shows higher methylation levels.

Fig. S5: Visualization of DMR associated DEGs. (A) Heatmap representing differential gene expression and differential methylation. Scale at the bottom of the heatmap represents log2 fold change and methylation level difference (MLD); blue indicates down regulation and hypomethylation whereas pink and yellow indicates up regulation and hypermethylation respectively. Gene ontology enrichment plot of DEGs associated with (B) CG-DMRs and (C) CHH-DMRs in SSc/s and STc/s genotypes under salinity stress. (D-E) IGV view of DMRs associated DEGs functionally enriched under salinity stress response in (D) SSc/s and (E) STc/s genotype. Each region includes the position of methylated cytosines (CG-purple, CHG-teal and CHH-olive green), differentially methylated regions (DMR) and gene expression of control and stress samples in SS and ST genotype in the last four rows.

Fig. S6: Expression profiling of genes involved in DNA methylation pathway and correlation of methylation in different sequence context. (A) Heatmap showing expression of genes encoding DNA methyltransferases during salinity stress response (B) Heatmap showing expression of genes encoding demethylases, argonaute and RDRP in control (SS-C and ST-C) and stress samples (SS-S and ST-S) of SS and ST genotypes. Scale at the bottom represents Z-score (pink represents low expression, green represents higher expression). (C-D) Correlation plots for gene body (GB) and flanking CG, CHG and CHH methylation levels of DEGs harboring DMRs in gene body (C) and its flanking regions (D) in SSc/s and STc/s comparisons.

Fig. S7: Methylation and small RNA density of TEs (A) Fraction of smRNAs identified in response to salinity stress in control (SS-C ad ST-C) and stress (SS-S and ST-S) samples of SSc/s and STc/s genotypes. (B) Density of 24-nt smRNAs and (C) methylation densities of 24-nt smRNAs associated with CHG and CG context hypermethylated TEs in TE body and 2kb flanking regions under control and salinity stress. (D) Small RNAs density of 21-nt small RNAs in CHH hypermethylated TEs that are associated and not associated with smRNAs in control (SS-C and ST-C) and stress (SS-S and ST-S) samples of SS and ST genotypes. (E) Methylation densities of hyper methylated TEs of CWA and non-CWA context. associated or not associated with 21nt smRNAs. (F) The base frequency distribution of 24-nt smRNAs associated with hypermethylated TEs.

Supplementary Table details

Table S1: Details of bisulphite sequencing libraries generated in this study.

Table S2: List of differentially methylated regions identified in SSc/s and STc/s comparisons in CG, CHG and CHH context.

Table S3: Total number of raw reads generated, high quality reads and mapping percentage of RNA sequencing data in control and stress samples of SS and ST genotype.

Table S4: List of differentially expressed genes identified in SSc/s, STc/s and SSc/STc comparisons.

Table S5: GO-enrichment analysis of commonly upregulated and downregulated genes in both SS and ST genotypes under salinity stress.

Table S6: List of DMR associated DEGs (dmDEGs) in in SSc/s and STc/s comparisons**.**

Table S7: Total number of 21-nt and 24-nt small RNAs identified in control and stress samples of SS and ST genotype in response to salinity stress.

Table S8: GO-enrichment analysis of SS and ST specific dmDEGs in CG and CHH context.
